# The Cottage Hospital in Fiction

**Published:** 1912-01-27

**Authors:** 


### THE COTTAGE HOSPITAL IN FICTION.

It is regrettable that those with the necessary intimate knowledge do not oftener see and exploit the literary side of institutional work. It has been very uncommon to find a medical officer or a secretary turning to his own and the public advantage the great human interest which is associated so obviously with hospital life. Good books about hospitals are much rarer than good books about schools—the one type of institution which has found its life ably reflected in fiction. Yet we have constant proofs that the doctor, in the case of books of travel, for instance, can produce delightful literature. But the field of hospital life and actualities has been neglected almost entirely by the profession, with the two following results.

First, even the doctor is not very well treated by the novelist; the frequency with which members of the medical profession are placed in an unfavourable light by writers of fiction is a somewhat curious circumstance. Under democratic governments the position of the medical man appears to be better than in monarchical countries; the subordinate position of the Court physician in the past, is not materially improved even to-day.

Secondly, though novelists are perhaps not so often found tilting against hospitals—probably through lack of accurate knowledge—yet the hospital, like the convent, tempts the sensation-monger to rush in and to emphasise, by his melodramatic fancies or fallacies, the real gap there is in modern literature. Still if hospital men will continue to write about every other subject but their own life and work, we suppose they must not be too hard on the adventurers of fiction.

Of late years several novels dealing with hospital incidents have appeared; unfortunately none has been of any literary merit, but the distorted impressions likely to be gained by the lay reader are all the more to be regretted by reason of the apparent plausibility—in the total absence of the correct picture—of some of the authors. An example—fortunately an extreme case—is now at hand in the shape of a recent novel entitled " In a Cottage Hospital,"* Its author ambitiously expresses the hope that his book will open the eyes of the public to the " goings-on " in the smaller hospitals of this country. This practically anonymous contribution to sensational fiction is asserted to be put forth with the object of bringing about tremendous reforms in hospital management. The author's actual achievement amounts to an extensive two-shillingsworth of very unpleasant sensationalism; there is quite a large number of closely printed pages for the money. Fortunately only the very ignorant will regard his story as in any degree approaching the possible, and from neither his matter nor his style can we induce ourselves to believe that the author is or ever was a fully qualified " practitioner with an extensive provincial practice."

That the book shows signs of being put together by one who has the sort of acquaintance of hospital life that would be expected from a probationer who was rejected after six weeks as unsuitable, we are prepared to admit, but the pious expressions in the preface are simply so much bunkum—if we may be forgiven the term. The reforming novelist, Dickens, or Charles Reade, would have been the first to see through them. No responsible person who has come in contact with the work of even the most benighted cottage hospital could have the impudence seriously to pretend that the scenes and the characters portrayed in this book represent a condition of affairs even exceptional, let alone normal, as the author implies. If they did, at a hint from the author the local paper would have been charmed to take it up; but where would the author's copyright have been then? For one thing, the very hospital in which the ludicrous tragedies are staged does not from its apparent size come under the recognised category of a " cottage " hospital at all.

The plentiful errors and absurdities of this romance can afford no amusement to those resident medical officers who might take the trouble to read it. The story is so thoroughly overdone that hospital managers may safely feel the threatened revolution long overdue, and leave this vulgar story for the delectation of that foolish class of reader whose facial types are so accurately portrayed upon its cover. We mention this objectionable coloured illustration not because it is so obviously a silly caricature of the two professions it has tried to illustrate, but because such imaginary types mirror correctly the type of reader which the author evidently had in mind. This book is so bad in every sense of the word, being mere vulgar fiction of the most trashy kind without justification or reason, that we wonder it has obtained the publicity of print.

* *In a Cottage Hospital.* London. Price 2s.

